# Android malware classification based on random vector functional link and artificial Jellyfish Search optimizer

**DOI:** 10.1371/journal.pone.0260232

**Published:** 2021-11-19

**Authors:** Emad T. Elkabbash, Reham R. Mostafa, Sherif I. Barakat

**Affiliations:** Information Systems Department, Faculty of Computers and Information Sciences, Mansoura University, Mansoura, Egypt; Torrens University Australia, AUSTRALIA

## Abstract

Smartphone usage is nearly ubiquitous worldwide, and Android provides the leading open-source operating system, retaining the most significant market share and active user population of all open-source operating systems. Hence, malicious actors target the Android operating system to capitalize on this consumer reliance and vulnerabilities present in the system. Hackers often use confidential user data to exploit users for advertising, extortion, and theft. Notably, most Android malware detection tools depend on conventional machine-learning algorithms; hence, they lose the benefits of metaheuristic optimization. Here, we introduce a novel detection system based on optimizing the random vector functional link (RVFL) using the artificial Jellyfish Search (JS) optimizer following dimensional reduction of Android application features. JS is used to determine the optimal configurations of RVFL to improve classification performance. RVFL+JS minimizes the runtime of the execution of the optimized models with the best performance metrics, based on a dataset consisting of 11,598 multi-class applications and 471 static and dynamic features.

## 1. Introduction

Worldwide, Android is the most common operating system (OS) with 87% of the OS market share as of 2021, with 1.6 billion users [1]. As mobile smart devices have grown in popularity, the number of mobile applications (apps) has grown exponentially. According to current reports, there are currently 3.04 million apps available for download in the Google Play store, surpassing 1 million apps in July 2013 [2].

Attackers, or those who wish to target users for malicious or nefarious purposes, capitalize on this broad distribution by exploiting many vulnerabilities present in the Android OS [3], including those pertaining to web views, dirty unstructured supplementary service data, Android secure socket layer/transport layer security, Android near-field communication, social and sharing authentication flaws, and zygote sockets and repackaging.

The use of machine-learning algorithms for Android malware detection can provide robust and efficient identification of these kinds of intentional obfuscations and harmful behaviors. However, machine-learning-based classifiers have two main problems. First, they must extract the feature-vector representations of the application; second, the time required for modeling is costly. To address the first problem, we gathered a heterogeneous feature set consisting of two analysis types: static and dynamic. To address the second problem, we used a feature selection process with an optimizer for the classifier.

Static analysis entails examining executables without performing an actual execution. In contrast, dynamic analysis involves running the executable and observing its results. Each has their own strengths and limitations; however, they work best when used together. Static analysis is fast, but malware can be disguised and therefore, may go unnoticed if the malware uses code obfuscation techniques. In contrast, these disguise techniques, along with polymorphic malware, have little impact on dynamic analysis, because the runtime execution is continuously monitored and analyzed. However, newly developed malware strains rapidly outpace traditional malware detection and analysis [4].

Feature selection enhances classification performance by removing redundant and irrelevant features from the dataset. The goals of feature selection are to reduce storage-space utilization and training time while still identifying the root issue at hand [5], because mobile-device hardware is limited. Many metaheuristic optimization algorithms currently optimize their feature selection processes, such as particle swarm optimization (PSO) [6], grey-wolf optimization (GWO) [7], and genetic algorithms (GAs) [8].

Random vector functional link (RVFL) networks are randomized functional-link neural networks. The input layer’s fundamental weight values provided to the hidden layer can be randomly created in the appropriate domain and retained during the learning process to avoid becoming stuck in a local minimum. RVFL applications are used to optimize many scientific areas applications, including performance predictions of solar photovoltaic thermal collectors [9], crude-oil price forecasting [10], and tensile behavior prediction of dissimilar friction stir-welded aluminum alloy joints [11].

Metaheuristic optimizers that use machine learning approaches work better than classical models; however, the machine learning approaches can still suffer from problems of overfitting and parameter optimization. Recent research [12, 13] has introduced hybrid models to improve the forecast accuracy of these models, while reducing the drawbacks associated with solo models.

We summarize our contributions as follows:

1. We use the artificial jellyfish swarm (JS) optimizer to select the optimal features of Android malware datasets, as illustrated in Section 4.2.
2. We provide an improved RVFL artificial JS optimizer algorithm (i.e., RVFL+JS), as shown in Section 4.3, to classify and categorize Android malware.
3. We compare RVFL+JS with RVFL+PSO, RVFL+GWO, RVFL+GA, and the standard RVFL model.
4. We compare the proposed RVFL+JS with recent Android malware detection studies.

The rest of this paper is organized as follows. In Section 2, recent related work on malware detection is discussed. Section 3 discusses the RVFL network, the artificial JS optimizer, the Android framework, and its applications for feature analysis. In Section 4, the proposed approach is introduced. Section 5 presents the performance metrics, results, experimental results, and a discussion of our findings. Finally, the conclusion and future works are provided in Section 6.

## 2. Literature review

Based on the enormous demand for Android devices, many studies have been conducted to provide a means for detecting Android malware and helping users deal with the spread of malware in their devices.

Sun et al. [14] provided an extreme learning-machine (ELM) approach to identify malicious Android applications by utilizing application attributes (e.g., permissions and application programming interface [API] calls) and employing an automated testing tool (i.e., WaffleDetector). Their approach showed good detection accuracy, short detection times, and required minimal human involvement.

Alternatively, Sulaiman et al. [15] provided a methodology that utilized the whale optimization algorithm (WOA) for feature selection of permission-based features in Android applications to increase their classification accuracy. Their results demonstrated improved accuracy over the state-of-the-art detection models that used WOA without feature selection.

J. D. Koli [16] presented a machine-learning-based malware detection system whose classifiers were trained using samples of benign and malicious applications with programmed features. When an application was run on the system, the system extracted the user permissions, vulnerable API calls, database details, and information about the dynamic, reflective, native, and cryptographic codes of the application. The extracted features were then used to train various machine-learning classifiers. Multiple experiments were conducted using Randroid, an Android malware detection method that uses random machine-learning classifiers, to verify system performance. He found that Randroid can reach 97.7% classification accuracy.

A machine-learning technique based on an evolutionary GA was proposed [17] for malware detection. Selected features gathered by the GA were applied to a machine-learning classifier to train it to identify malware before and after feature selection. The results demonstrated that the GA provided the best feature subset that led to an approximate 50% reduction in feature dimensions.

Kim et al. [18] suggested a framework that incorporated many static features to represent various Android applications. Features were enhanced using a feature extraction approach to identify malware in real-world conditions. Multimodal deep learning was also employed in the malware detection model.

Türker and Can [19], alternatively, introduced a classification approach to categorize Android malware by family. Their algorithm used static features to detect malware by utilizing many different machine-learning classifiers, including support vector machines (SVMs), decision trees (DTs), logistic regression (LR), k-nearest neighbors (KNNs), random forest (RF), majority voting, multi-layer perceptrons (MLPs), and AdaBoost. The tested machine-learning models yielded high accuracy in categorizing malware families, demonstrating the utility of the extracted features. The SVM classifier had the most significant impact, with an overall 98.86% 10-fold accuracy.

BadHani et al. [20] developed methods of classifying Android apps in a binary manner as either benign or malicious based on their static features. They employed five single ML classifiers and three feature sets. In their first experiment, classifiers (i.e., DT, ELM, LR, SVM, and a repeated incremental pruning tool to produce error reduction) delivered improved results on some performance metrics. Ensemble learning was further refined in their second experiment to improve performance further.

Waleed Ali [21] presented an Android malware detection approach to improve SVMs with evolutionary boundary algorithms to boost Android malware detection. PSO and GA tools (i.e., DroidHESVMGA and DroidHESVMPSO) handled their optimization problems to enhance the SVM performance and improve the precise detection of Android viruses. The testing accuracy of Droid-HESVMGA was 96.9%, whereas that of Droid-HESVMPSO was 96.0%.

Mehtab et al. [22] created AdDroid, which analyzes and detects fraudulent behavior in Android applications by leveraging rules that comprise distinct combinations of items. Each rule depicts an Android application’s particular behavior and simulates the execution of various device tasks via Bluetooth. To train a model capable of identifying malicious applications based on the static analysis of Android applications, AdDroid used an ensemble-based machine-learning technique in which Adaboost was paired with classifiers. Feature selection and extraction procedures were utilized to provide the most specific rules. A dataset of 1,420 Android apps containing 910 malicious and 510 benign apps was used to create the model.

Zhu et al. [23] created the SEDMDroid framework to identify Android malware using an upgraded deep-learning stacked ensemble technique. This dual-layered classifier architecture used an MLP classifier on the first tier and an SVM fusion classifier on the second. Furthermore, the design incorporated a double disturbance method, in which sample and feature spaces were disturbed to guarantee accuracy and variety of their technique in its base classification. A multi-level static-feature dataset was used to evaluate their technique.

Mahdavifar et al. [24] suggested an effective and efficient Android malware category classification system that used a semi-supervised pseudo-label deep neural network. Although there were very few labeled training datasets with which they could train their system, their approach performed better than deep-neural networks that used supervised learning. Their dataset contained a 11,598 multi-class application library with hybrid features across all five malware categories (i.e., adware, banking, short message service (SMS), riskware, and benign). With a specific number of hidden layers and hidden neurons, their method achieved 96.7% accuracy in detecting malware.

Al-Fawa’reh et al. [25] introduced a convolutional neural-network-based approach for malware detection using hacked Android package files (APKs). By leveraging different sets of balanced and unbalanced datasets from those created by [24], the authors showed that their method was highly accurate at detecting malware, with an overall accuracy of 96.4%. Additionally, the transfer-learning models saved training time relative to comparison models.

Additionally, previous research [26] used an RF algorithm based on the datasets created by [24] to achieve high accuracy in the classification of banking malware. The authors used the CICFlowMeters tool to obtain the required comma-separated-value files from the malware for use in malware detection. The classification results using the RF algorithm with feature selection was 92.5%, and a precision value of 93.28% was achieved with a recall of 93.73%.

Taken together, this review of prior work demonstrates that deep-learning models used for Android malware classification can produce excellent results when the application features are utilized. However, even better outcomes are possible if better feature extraction techniques are used with GAs [17]. Therefore, in this paper, computational models were used to classify Android malware from the hybrid features of applications using a feature selection technique.

## 3. Methods

### 3.1. Android operating system

Since Google deployed Android in 2005 [1], the Android OS has become the dominant market platform for mobile operating systems, with 1.6 billion active Android devices representing 74.13% of mobile devices worldwide and a total of 3.04 million applications on the official market (Google Play) as of 2020. Thus, the Android OS is a highly valued target for malware developers.

The Android OS provides a collection of software components built around the Linux kernel. Thus, it is open-source, making it popular for both developers and consumers. It runs primarily on mobile devices and tablets, although recently it has also been used to run many internet-of-things (IoT) devices, such as televisions, washing machines, home appliances, and cars. The open-source nature of Android serves its users’ needs, it may also endanger user privacy, owing to the permissions that are freely granted to access sensitive information.

#### 3.1.1. Application static analysis

Static analysis [27] typically requires the input of a program’s source code, but this allows the investigation of said code without running it, and thereby causes exposure to potential threats. Functional results are obtained by checking or simulating the coding structure and statement sequences while handling variable values throughout the code’s various functions, permissions, and API calls.

Furthermore, static analysis is performed in a non-runtime environment, whereas dynamic analysis is performed live. Thus, static analysis is good for functional testing, whereas dynamic analysis is best used to reflect the unique circumstances that cannot be satisfied with purely functional analysis [28].

#### 3.1.2. Application dynamic analysis

Dynamic behavioral analysis is defined as the detection and tracking of the behaviors of Android applications during runtime execution to determine the existence of malware categories [29]. Application-control actions include reading and writing files, monitoring incoming and outgoing network details, employing encryption operations, detecting information leakage, sending SMS messages, and making calls [30].

#### 3.1.3. Hybrid analysis

Hybrid analysis combines static and dynamic analysis methods to further examine the Android application source code while observing the application behaviors in real-time.

### 3.2. Artificial JS optimizer

The Jellyfish optimizer was introduced by Chou and Truong in 2021 [31]. This algorithm was inspired by the movement and search behavior of jellyfish in the ocean. The implementation of JS is based on the following three approaches [31, 32]:

1. The jellyfish obey only one dominating condition (i.e., the ocean current or the internal movement of a group) based upon a time-control procedure.
2. The jellyfish desire to be positioned near food quantities.
3. Food is allocated to jellyfish using a predefined fitness function.

When the jellyfish move inside a swarm, a *bloom* is created as the result of either active or passive movements. Food quantities vary with jellyfish movement along a food-search path. After comparisons between food quantities, the best value of the fitness function estimates the best locations. The different steps in the JS optimizer can be described as follows.

The population is initialized utilizing a logistic map [33]:

$${\vec{P}}_{i+1}=\vartheta .{\vec{P}}_{i}\left(1-{\vec{P}}_{i}\right),0\leq P_{0}<1,$$ (1)

where ${\vec{P}}_{i}$ is the logistic value of the *i*^th^ jellyfish position. Good performance is achieved if the *ϑ* value is equal to four, as proposed in [31]. The ocean current is mathematically described as

$${\vec{P}}_{i}\left(s+1\right)={\vec{P}}_{i}\left(s\right)+{\vec{rand}}_{1}*\left({\vec{P}}^{*}-\gamma *{rand}_{2}*\mu \right),$$ (2)

where ${\vec{rand}}_{1}$ denotes the trajectory random numbers between 0 and 1, * is the vector multiplication operator, *γ* represents the distribution coefficient, *rand*_2_ is a random number between 0 and 1, and *μ* denotes the population average.

The movements of the jellyfish are controlled by active and passive motions. A motion is designated as passive if the jellyfish move within the current. Hence, the new position is described by the following equation:

$${\vec{P}}_{i}\left(s+1\right)={\vec{P}}_{i}\left(s\right)+{rand}_{3}*\rho *(X_{b}-Y_{b}),$$ (3)

where *rand*_3_ represents a random number between 0 and 1, *ρ* is the movement distance from the current position, and *X*_*b*_ and *Y*_*b*_ denote the upper and lower bounds of the search space, respectively. The new position is presented in a continuous form. Active (intentional) motion is defined as:

$${\vec{P}}_{i}\left(s+1\right)={\vec{P}}_{i}\left(s\right)+{\vec{rand}}_{1}*\vec{M},$$ (4)

where $\vec{M}$ represents the direction of movement expressed in the following equation:

$$\vec{M}=\left\{\begin{array}{ll} {\vec{P}}_{i}\left(s\right)-{\vec{P}}_{j}\left(s\right),if\;fitness\;function\left({\vec{P}}_{i}\right)<fitness\;function\left({\vec{P}}_{j}\right), & \left(5\right)\\ {\vec{P}}_{j}\left(s\right)-{\vec{P}}_{i}\left(s\right),otherwise. & \left(6\right) \end{array}\right.$$

The ocean current, as well as active and passive motions, are alternated using the time-control procedure, *C*(*s*). This procedure is mathematically expressed as

$$C\left(s\right)=\left(1-\frac{s}{S_{max}}\right)*(2*{rand}_{1}-1).$$ (7)

It can be observed that as time proceeds, each jellyfish continues to move inside the swarm to find the best food location.

The main steps of the artificial JS optimizer algorithm are shown in Algorithm 1.

Algorithm 1: JS optimizer algorithm

**artificial JS optimizer**

**Input ← objective function *f*(*P*), population size (N**_**pop**_ **), search space [X**_**b**_ **:Y**_**b**_ **], Max number of iterations (Max**_**int**_ **)**

**Output ← the best results and visualization (jellyfish bloom)**

1. ** Begin**

2. **   Define objective function F(P)**

3.**   Set the search space, population size N**_**pop**_

4.**   Max number of iterations Max**_**int**_

5.**   Initialize population of jellyfishes xi**

6.   **Calculate the food at each location**

7.   **Find jellyfish with the best location**

8.   **Initialize time: s = 1**

9.   **while s < Smax**

10.    **Fitness evaluation of each iteration (solution)**

11.    **For i = 1:N**_**pop**_

12.     **Calculate the time control, C(s), using** Eq (7)

13.     **If c(t) > = 0.5: jellyfish follows ocean current**

14.     **else: Jellyfish moves inside a swarm**

15.      **If Rand [0:1]> = 1-C(s): jellyfish moves passively**

16.       **else: jellyfish moves actively in its direction**

17.      **End if**

18.     **End if**

19.    **End for**

20.    **Update new position to jellyfish**

21.    **Check new bound condition**

22.    **Check stop condition**

23.    **Output the best results and visualization (jellyfish bloom)**

24.   **End while**

25.  **End**

### 3.3. RVFL network

Because of their universal approximation capabilities, single-layer feedforward neural networks are commonly used to solve classification and regression problems [34].

**Fig 1:**
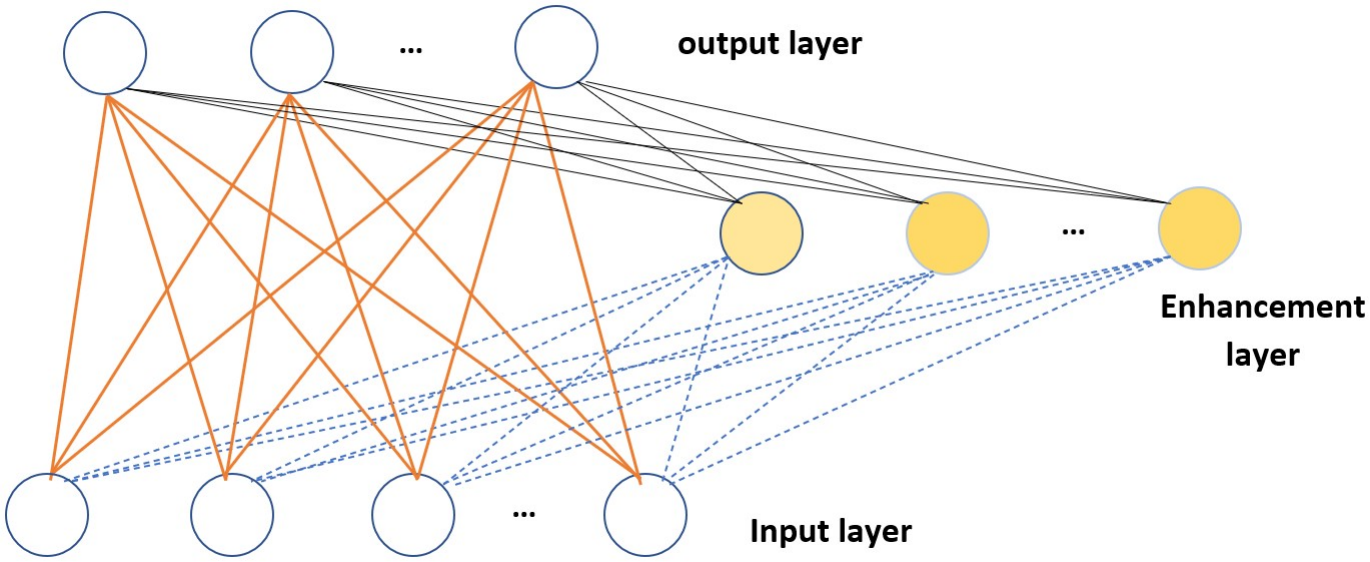
Structure of the randomized vector functional link network. The white circles represent input- and output-layer nodes, whereas the yellow circles represent enhancement-layer nodes.

RVFL networks [35] are created when real weight values from the input layer to the hidden layer are randomly created in the appropriate domain and are subsequently retained during the learning process to avoid the local minima problem [36]. Fig 1 shows the structure of an RVFL network.

The RVFL network randomly initializes fixed weights between the input nodes and the enhancement layer in the range [-F, +F]. F is a measurement factor to be calculated for each dataset during the parameter-tuning stage. During training, only the output weights are changed, and they are calculated using the Moore–Penrose pseudo-inverse and ridge regression method.

Enhancement nodes convert input features into enhanced features. First, the input weights and node biases are generated randomly. Then, both the original and the enhanced features are concatenated and assigned to output neurons in the output layer.

Let the input layer of the RVFL network receive a group of labeled data, {(*x*_*i*_, *y*_*i*_) | *x*_*i*_ ∈ *R*^*n*^, *y*_*i*_ ∈ *R*^*n*^, *y*_*i*_ ∈ *R*^*m*^, *i* = 1, …, *N*}; then, the output of the j^th^ enhancement node is calculated as:

$$D_{j}\left(a_{j}x_{i}+b_{j}\right)=\frac{1}{1+e^{-\left(a_{jx+b_{j}}\right)}},$$ (8)

where *a*_*j*_ ∈ [−*F*, *F*] and *b*_*j*_ ∈ [0, *F*] are the weight and bias, respectively, between the input node and the enhancement layer. The RVFL output is:

Y=Hw, (9)

where *w* ∈ *R*^*n*+*p*^ represents the weight of the output, and H is an input data matrix. The enhancement node output D is

$$H=\left[H_{1}H_{2}\right]$$ (10)

$$H_{1}=\left[\begin{array}{ccc} x_{11} & \ldots & x_{1n}\\ \vdots & \ddots & \vdots \\ x_{N1} & \ldots & x_{Nn} \end{array}\right],H_{2}=\left[\begin{array}{ccc} D_{1}\left(a_{1}x_{1}+b_{1}\right) & \ldots & D_{P}\left(a_{P}x_{1}+b_{P}\right)\\ \vdots & \ddots & \vdots \\ D_{1}\left(a_{1}x_{N}+b_{1}\right) & \ldots & D_{P}\left(a_{P}x_{N}+b_{P}\right) \end{array}\right]$$

The weight, w, is calculated using the ridge regression:

$$w={\left(H^{T}H+\frac{I}{C}\right)}^{-1}H^{T}Y$$ (11)

or using the Moore–Penrose pseudo-inverse:

$$w=H^{†}Y,$$ (12)

where †, I, and *C* represent the Moore–Penrose pseudo-inverse, identity matrix, and tradeoff parameter, respectively.

## 4. Proposed approach

**Fig 2:**
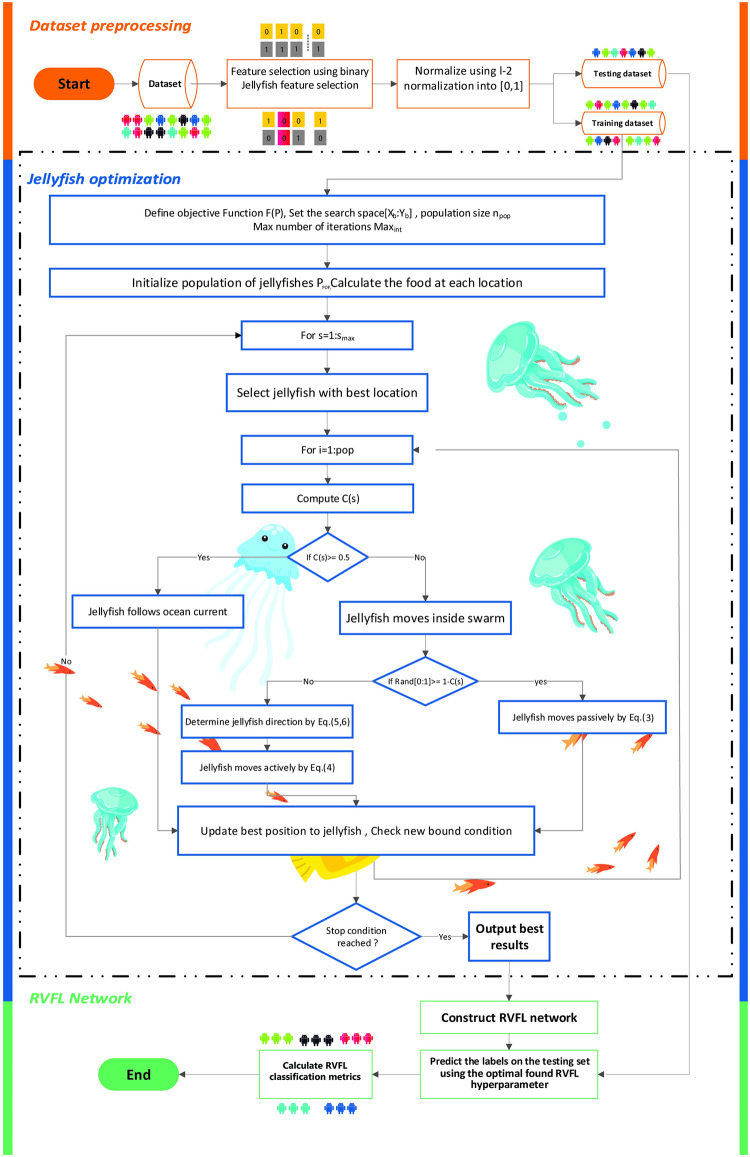
Proposed randomized vector functional link plus artificial jellyfish swarm model.

Many studies have been published on the use of deep-learning classifiers to detect Android malware from application feature vectors. Fig 2 illustrates the structure of our approach. RVFL+JS is divided into three stages. The first stage preprocesses the essential dataset features and normalizes the feature frequencies to within [0,1]. During the second stage, the artificial JS optimizer is launched to work with the final step to predict the best hyperparameters of the RVFL network where the classifications are modeled [37].

### 4.1. Dataset acquisition

We used a public online dataset [24] from the University of New Brunswick Canadian Institute for Cybersecurity website (https://www.unb.ca/cic/datasets/maldroid-2020.html).

### 4.2. Dataset normalization

After obtaining the dataset [24] as a CVS file containing vectors of features of size 470 for each application, as extracted by the dataset authors, we configured and defined each feature with the frequency of invoking all distinct behaviors of all APK files at a low level. The characteristics vectors were normalized into [0,1] values via *ℓ*_2_ normalization, which scales each vector to the square root of the sum of the squares of all values. The vector’s *ℓ*_2_-norm = 1. Let *y* = (*y*_1_, *y*_2_, *y*_3_ ⋯, *y*_*n*_) be a vector in the n-dimensional real vector space, $R^{n}$; then, the *ℓ*_2_-norm of vector *y*, denoted by |*y*|, is defined as $|y|=\sqrt{y_{1}^{2}+y_{2}^{2}+y_{3}^{2}+\cdots +y_{n}^{2}}$.

### 4.3. Feature selection using artificial JS optimizer

Feature selection is generally regarded as a preliminary stage in which the optimal subset of features is determined from the collection of all features. Because our work on Android devices is limited by the available hardware, our platform’s goal was to decrease complexity by ignoring extraneous (i.e., redundant) features to boost the machine-learning model’s prediction accuracy.

**Fig 3:**
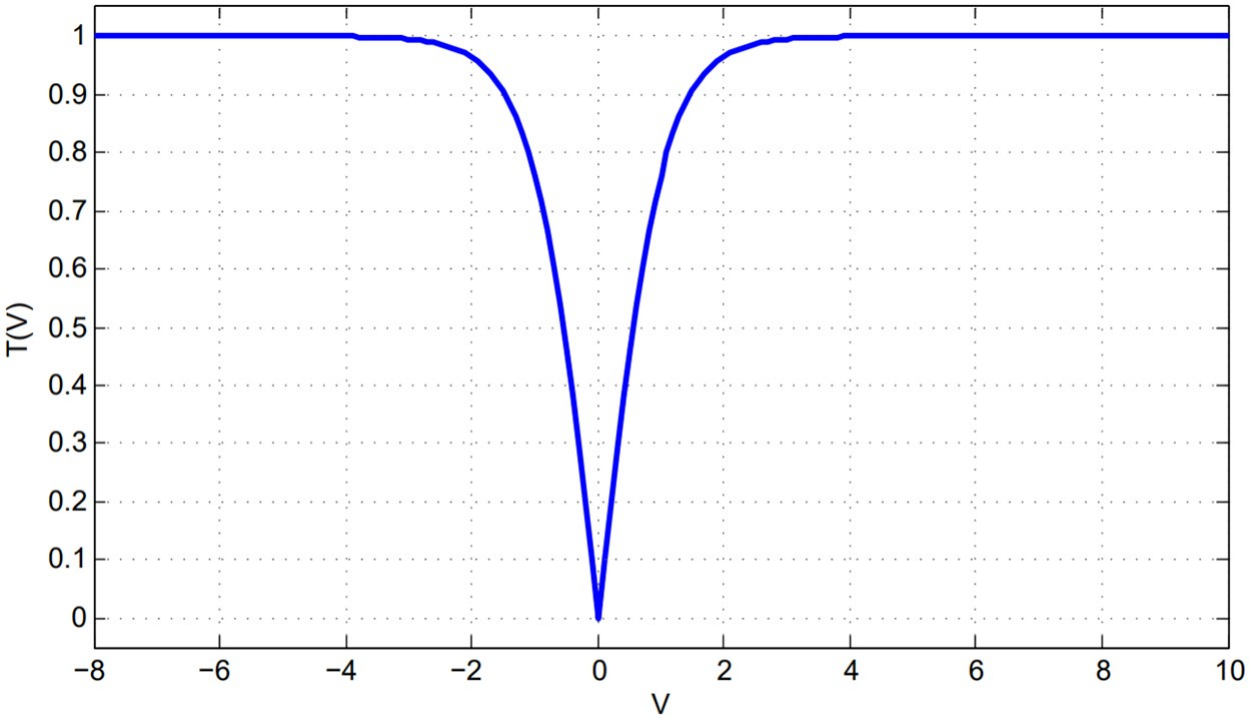
V-shaped transfer function.

Because feature selection optimization works as a binary problem using a search space that is shaped as a hypercube, the position vector uniquely identifies a specific location within the search space. In a moving system, the current position vector is added to the step vector to obtain the new position. This technique must be modified to address binary optimization concerns. The continuous data are translated into binary using a V-shaped transfer function [38], as shown in Fig 3.

The value of the *d*^th^ dimension of the *i*^th^ step vector in the current iteration (*t*) is used as an input to Eq (13) to generate the probability of changing that element to 0 or 1.


$$T\left(v_{d}^{i}(t)\right)=|\left(v_{d}^{i}\left(t\right)\right)/\sqrt{1+{\left(v_{d}^{i}\left(t\right)\right)}^{2}}|$$ (13)

The *i*^*th*^ element of the position vector is converted to 0 or 1 using Eq (14) by plugging the outcome $T\left(v_{d}^{i}(t)\right)$ obtained from Eq (13).

$$X(t+1)=\left\{\begin{array}{cc} -X_{t} & r<T\left(v_{k}^{i}(t)\right)\\ X_{t} & r\geq T\left(v_{k}^{i}(t)\right) \end{array}\right.$$ (14)

Where *r* is a function that generates a random number between 0 and 1. The value of *r* has a major role in determining whether the value of *X*_*t*_ is flipped. When the value of $T\left(v_{k}^{i}(t)\right)$ is small, the chance of flipping the new value *X*(*t* + 1) will be also small.

The jellyfish with highest fitness value in each iteration is considered as the best location. The best location jellyfish and the jellyfish selected by the selection mechanism that searches for food and a time control mechanism guide the movement of the jellyfish. Because this solution is continuous, as illustrated in section (3.2), it must be converted to a binary version using a V-shaped transfer function to suit the feature selection problem. Additionally, the transfer function should supply a significant possibility to change its position for a large absolute velocity value, because it would be far from the best solution.

In RVFL+JS, the solution is a one-dimensional vector of *Feat* elements where *Feat* is the number of features in the original dataset. A cell value of 1 or 0 is attached to each vector element. When the associated feature is selected, the value is set to 1., otherwise the value is set to 0.

For feature selection, the solutions are represented in binary form, either bit 1 or 0. Basically, bit 1 denotes the selected feature, while bit 0 represents the unselected feature. For example, given some features subset of 10 dimensions (1,1,0,1,0,1,1,0,1,1), the 3^rd^, 5^th^, and 8^th^ features are not selected and the others are used for learning phase.

A critical component to consider when planning any optimization procedure is the objective function. As a wrapper technique, feature selection attempts to retain minimal features while maximizing the accuracy of the learning algorithm. Both the selection ratio (minimization) and the classification error rate (minimization) are targeted in this study with the following objective function:

$$\text{Fitness}\;\text{function}=\alpha \times ER\left(M\right)+\beta \times \frac{\left|Sfeat\right|}{\left|Feat\right|},$$ (15)

where *ER(M)* represents the classification error rate when using the KNN classifier, |*Sfeat*| is the selected features count, |*Feat*| is the original features count, and *α* and *β* are parameters in the interval [0, 1], where *α* is the complement of *β*. Value *α* represents the weight of the classification error rate, and *β* represents the selection ratio.

The dataset was randomly divided into a training dataset representing 80% of the data, and the validation dataset was the remaining 20%. The classification error was computed using the KNN with k = 5. A KNN-based model was chosen because of its simplicity, ease of implementation, and inexpensive comparison computation [39].

### 4.4. Classification using RVFL+JS

As mentioned in Section 3.2, JS optimizes the RVFL network to determine the best hyperparameters and to provide the highest classification accuracy rate. For this reason, the dataset was divided into training and testing datasets. First, the training dataset was processed during the RVFL network training stage, followed by testing to determine model performance. The full classification process is performed according to the following procedures:

- **Initialization**: The JS begins by randomly generating *npop* for the optimization process; each population is a vector of the hyperparameter from a specific range of RVFL hyperparameters.
- **Fitness evaluation**: The second procedure determines the fitness value of the population by constructing the RVFL network and training it using the JS parameters and the training dataset. The construction of the RVFL+JS is achieved by extracting the necessary elements from the population. Many neurons, biases, scale modes, scales, and seeds are used to construct the RVFL.
- When the jellyfish move substantially inside a swarm, a *bloom* is created. The movements are either active or passive. The food quantity varies at different locations according to the path of the swarm during the food search. After comparisons of food quantity, the best value of the fitness function estimates the best locations.
- The active and passive motions control the movements of the jellyfish using the time control procedure, *C*(*s*), as time proceeds. Thus, jellyfish continue to move inside the swarm to attempt to find the best location for food.
- **Termination**: All previous procedures, apart from initialization, are repeated for as many iterations as necessary. Then, the best solution returned by the RVFL+JS is used to test the model’s accuracy against the testing dataset.

## 5. Experimental results

### 5.1. Metrics

The performance metrics to evaluate and compare the algorithms combined with the RVFL network include accuracy, sensitivity, specificity, precision, false-positive rate (FPR), and F1-score [40].

Because we performed our classification on a multi-class dataset, we calculated performance metrics as:

- A true-positive (TP) value was found when the actual and predicted values were the same.
- A true-negative (TN) value for a class was the sum of values of all columns and rows apart from the values calculated.
- A false-positive (FP) value for a class was the sum of values for the corresponding column, apart from the TP value.
- A false-negative (FN) value for a class was the sum of the values of corresponding rows, apart from the TP value.

The accuracy of the model was defined as the number of correct predictions. In common usage, this is compared to all the previous predictions:

Accuracy=(TP+TN)/(TP+FN+FP+TN) (16)

Sensitivity is the screening test’s ability to find a TP. This metric measures the uncertainty in the output of the model [41]:

Sensitivity=TP/((TP+FN)*100) (17)

Specificity is the screening test’s ability to find a TN:

Specificity=TN/((FP+TN)*100) (18)

Precision indicates how many of the truly predicted values turned out to be positive:

Precision=TP/(TP+TN) (19)

The recall measure corresponds to the proportion of values predicted as positive that were actually positive:

Recall=TP/(TP+FN) (20)

Lastly, the F1-score is the mean of precision and recall on a harmonic scale. The macro-F1 is used for multi-class classifications [42], calculated using the previous metrics and the classes of the unweighted mean [41]:

F1-Score=(2*Precision*Recall)/(Precision+Recall). (21)

### 5.2. Dataset

Mahdavifar et al. [24] introduced a new Android malware dataset (CICMalDroid2020), which is advantageous to use owing to its four key properties:

- **Large:** It encompasses 11,598 Android apps.
- **Recent**: New (up to 2018) and advanced Android samples are included.
- **Diverse**: Samples consists of five different application families: adware, banking malware, SMS malware, riskware, and benign.
- **Comprehensive**: It contains hybrid features.

The results were analyzed and divided into three large groups:

- **Statically extracted information**: permissions, file types, intents, services, frequency counts for various file types, occurrences of obfuscation, and sensitive API invocations.
- **Dynamically observed behaviors**: system calls, binder calls, and composite behaviors.
- **Packet capture (PCAP):** traffic network logs reported during the study.

The dataset uses a multi-class five-category family grouping. The distribution is shown in Table 1.

**Table 1:** Dataset category distribution.

CATEGORY	NUMBER OF SAMPLES
**ADWARE**	1,253
**BANKING**	2,100
**SMS**	3,904
**RISKWARE**	2,546
**BENIGN**	1,795
**TOTAL**	11,598

### 5.3. Experimental results

To accommodate fair comparisons, experiments were carried out for all procedures under the same conditions. The main details of the hardware and software of the used computing system are listed in Table 2.

**Table 2:** Hardware and software of computing system.

Name	Settings
Hardware	Intel(R) Core (TM) i7-6700HQ CPU @ 2.60 GHz
Memory	8,192-MB RAM
Hard drive	128 SSD, 1 Tera HDD
Software	Windows 10 Pro 64-bit
Language	MATLAB R2020a

For RVFL Matlab coding, we used the same code from the standard RVFL literature provided publicly by the copyright holders [43], which ensured that RVFL+JS can be compared with past and future works. The only way to compare algorithms fairly was to initialize them to the same population sizes and to apply the same process termination conditions (i.e., number of iterations). Our code is available online in a GitHub repository for public use https://github.com/emadtawfeek/optimizing-RVFL-with-Jellyfish-search-algorithm. Table 3 lists the parameters of our model.

**Table 3:** Parameter settings of all metaheuristic algorithms for optimizing RVFL.

Parameter	
Population	50
Number of iterations	100

Table 4 lists private parameter settings of each metaheuristic optimization algorithm according to the relevant articles from which they were sourced [6–8, 31].

**Table 4:** Private parameter settings of each metaheuristic optimization algorithm.

PSO	Cognitive component (*c*_1_)	2
	Social component (*c*_2_)	2
	Inertial weight	0.2–0.9
GA	Selection	Stochastic Universal Sampling
	Crossover	uniform
	Mutation	Real coded
	Alpha α	0
	Crossover probability	0.9
	Mutation probability	0.1
GWO	Α	decreased from 2 to 0
JSA	The parameters governing the JS algorithm include population size and number of iterations.	

Optimizing all RVFL hyperparameters can lead to an NP-hard problem. Hence, we used just this set of hyperparameters to be optimized. We set a search space for each algorithm to obtain the best combination of hyperparameters to optimize the RVFL network, as illustrated in Table 5.

**Table 5:** Lower and upper space-search bounds for all metaheuristic algorithms.

	N	Bias	Scale	Scale mode
Lower bound	100	0	0.0001	1
Upper bound	1,000	1	0.9999	3

Considering the above preconditions, all RVFL hyperparameters are listed:

- N: represents the number of hidden neurons
- Bias: checks whether the network has a bias in the output neurons
- Scale: percent of random features that will be linearly scaled
- Scale mode: illustrates how features will be scaled (1: features for all neurons; 2: features for each hidden neuron separately; and 3: scale the randomization range for a uniform distribution.)
- Seeds: random
- Activation Function: Radbas
- Updating Method: ridge regression.
- Link between the input and output: true
- Random Type: different randomization methods (currently only support Gaussian and uniform). We used uniform.

RVFL+JS obtained the optimized hyperparameters listed in Table 6. Then, we ran each algorithm for 30 times. We obtained the average of all runs, the standard deviation to measure the amount of variation or dispersion for the set of values, and the best execution result. Then, we compared our results on these different metrics for RVFL+JS with those of PSO, GWO, and GA. We then compared RVFL+JS with standard RVFL. The results are listed in Tables 6–11 for the training and testing datasets.

**Table 6:** Measurement accuracy of RVFL+ JS against other metaheuristic algorithms.

Accuracy	Training			Testing		
Algorithm	AVG	SD	Best	AVG	SD	Best
RVFL + JS	**97.58%**	0.5278%	**98.15%**	**97.22%**	0.4241%	**98.41%**
RVFL + GA	92.01%	0.5229%	97.96%	96.89%	**0.3165%**	97.41%
RVFL + PSO	92.69%	**0.3392%**	97.91%	96.85%	0.5509%	**98.41%**
RVFL + GWO	92.93%	0.3935%	98.04%	96.95%	0.3797%	**98.41%**
Standard RVFL	89.36%	0.7056%	90.36%	89.03%	1.1050%	90.39%

**Table 7:** Measurement macro F1-score of RVFL+ JS against other metaheuristic algorithms.

F1-score	Training			Testing		
Algorithm	AVG	SD	Best	AVG	SD	Best
RVFL + JS	**97.90%**	0.4166%	**98.36%**	**97.51%**	0.4055%	**98.58%**
RVFL + GA	92.42%	0.4377%	98.21%	97.23%	**0.2892%**	97.77%
RVFL + PSO	93.11%	**0.2870%**	98.16%	97.21%	0.5017%	**98.58%**
RVFL + GWO	93.37%	0.3306%	98.28%	97.32%	0.3387%	**98.58%**
Standard RVFL	89.92%	0.7227%	90.99%	89.51%	1.2128%	90.81%

**Table 8:** Measurement sensitivity of RVFL+ JS against other metaheuristic algorithms.

Sensitivity	Training			Testing		
Algorithm	AVG	SD	Best	AVG	SD	Best
RVFL + JS	**97.72%**	0.5376%	**98.31%**	**97.29%**	0.4380%	**98.47%**
RVFL + GA	92.13%	0.4887%	97.96%	96.93%	**0.3563%**	97.51%
RVFL + PSO	92.83%	**0.3229%**	97.89%	96.88%	0.5587%	**98.47%**
RVFL + GWO	93.06%	0.3712%	98.06%	97.00%	0.4010%	**98.47%**
Standard RVFL	88.74%	0.7724%	89.80%	88.43%	1.1736%	90.11%

**Table 9:** Measurement specificity of RVFL+ JS against other metaheuristic algorithms.

Specificity	Training			Testing		
Algorithm	AVG	SD	Best	AVG	SD	Best
RVFL + JS	**99.33%**	0.1631%	**99.50%**	**99.23%**	0.2411%	**99.56%**
RVFL + GA	93.96%	0.1498%	99.42%	99.16%	0.1597%	**99.56%**
RVFL + PSO	94.67%	**0.0976%**	99.41%	99.11%	0.1541%	**99.56%**
RVFL + GWO	95.00%	0.1144%	99.45%	99.14%	**0.1062%**	**99.56%**
Standard RVFL	97.00%	0.2156%	97.27%	96.93%	0.3023%	97.35%

**Table 10:** Measurement FPR of RVFL+ JS against other metaheuristic algorithms.

FPR	Training			Testing		
Algorithm	AVG	SD	Best	AVG	SD	Best
RVFL + JS	**0.00677**	0.00132	0.01010	**0.00768**	0.00141	0.01240
RVFL + GA	0.00793	0.00150	0.01010	0.00884	**0.00071**	0.01010
RVFL + PSO	0.00798	**0.00098**	**0.00960**	0.00885	0.00154	0.01170
RVFL + GWO	0.00839	0.00114	0.01000	0.00857	0.00106	**0.00940**
Standard RVFL	0.03001	0.00212	0.03720	0.03069	0.00302	0.03850

**Table 11:** Execution time of RVFL+ JS against other metaheuristic algorithms.

Algorithm	Time (minutes)
RVFL + JS	**30.5**
RVFL + GA	40.1
RVFL + PSO	37.9
RVFL + GWO	62.3
Standard RVFL	0.02

### 5.4. Statistical analysis

In general, comparing algorithms using statistical metrics such as best, SD, and Avg over 30 independent runs does not compare each run. It is still possible for superiority to arise by chance despite the low probability in 30 runs. Thus, a non-parametric statistical test was used to compare the findings of each algorithm and to determine their significance.

The Wilcoxon rank sum test was employed to determine the significance of the data in this work as a non-parametric statistical test [44]. Table 12 summarizes the p-values at 5% derived from this test. Since p-values less than 0.05, Table 12 demonstrates RVFL + JS’s significant advantage to the other methods.

**Table 12:** Results from the Wilcoxon rank sum test (p ≥ 0.05).

	RVFL + JS vs. RVFL + GA	RVFL + JS vs. RVFL + PSO	RVFL + JS vs. RVFL + GWO	RVFL + JS vs. Standard RVFL
Training dataset	0.0174	0.0083	0.0018	2.8544e-18
Testing dataset	0.0344	7.0459e-04	7.8006e-04	8.0907e-19

### 5.5. Discussion and comparison

Feature selection using JS requires a wrapper methodology in which multiple feature sets are prepared, analyzed, and compared for better combinations. Predictive models were employed to determine which combinations of features best predict the model performance.

We used a dataset [24] of hybrid features containing intents, method tags, permissions, API calls, file types, obfuscation, and components for static analysis. We used system calls, binder calls, composite behaviors, API calls, networks, and logs for dynamic analysis.

**Fig 4:**
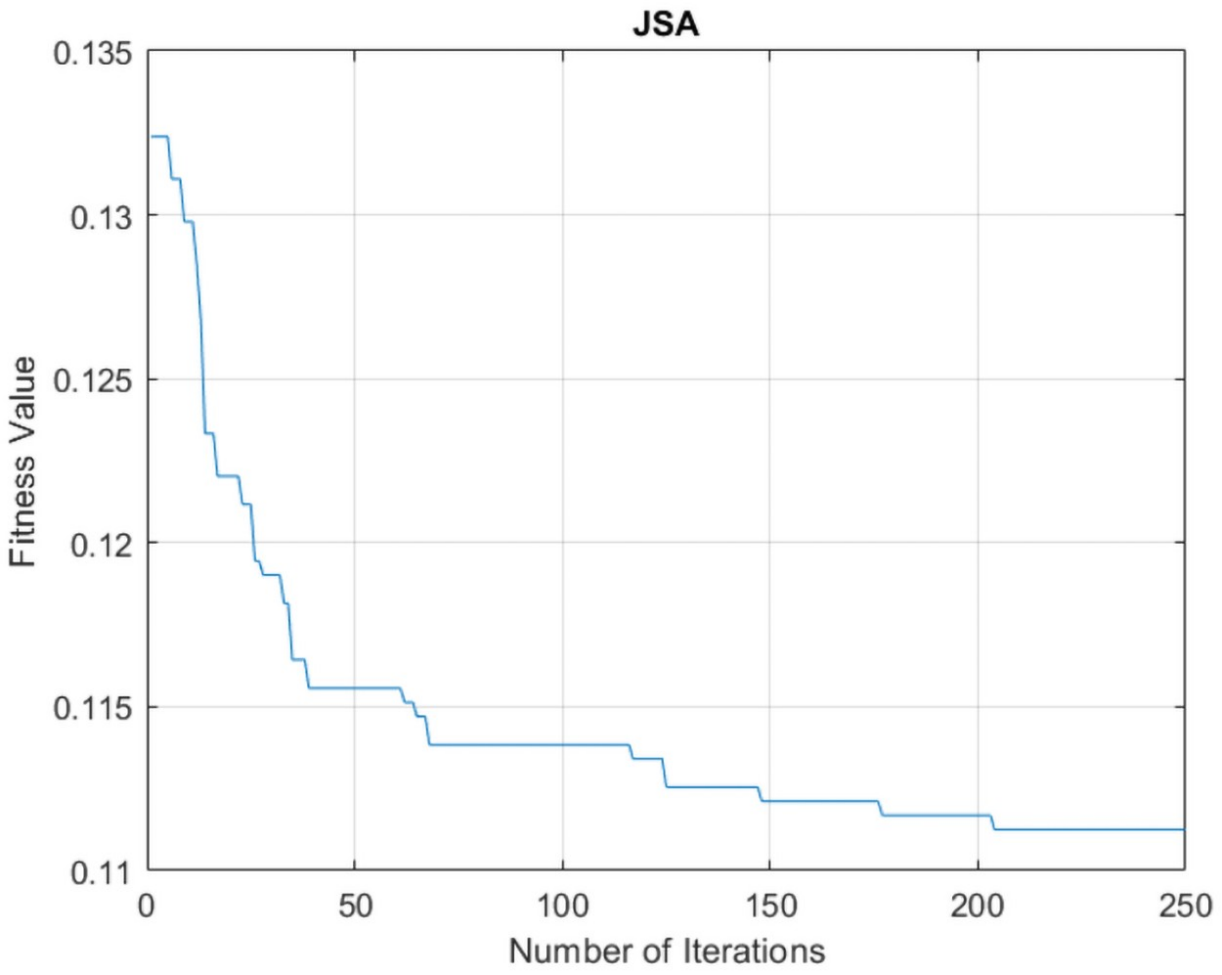
Convergence curve of feature selection process accuracy.

JS binary feature selection reduced the number of hybrid features from 470 to 195, and it required 8.5 hours, with 88.87% accuracy percentage. The run consisted of 250 iterations. Fig 4 illustrates the convergence curve of the feature selection process. The feature reduction percentage exceeded the percentage of publication [17], which reduced the dimensionality using the GA. However, the no-free-lunch optimization theorem showed that there was no assurance that the optimizer would be good enough to address all of the optimization problems. Thus, the current stochastic-based feature selection methods may not be suitable for some tasks.

**Fig 5:**
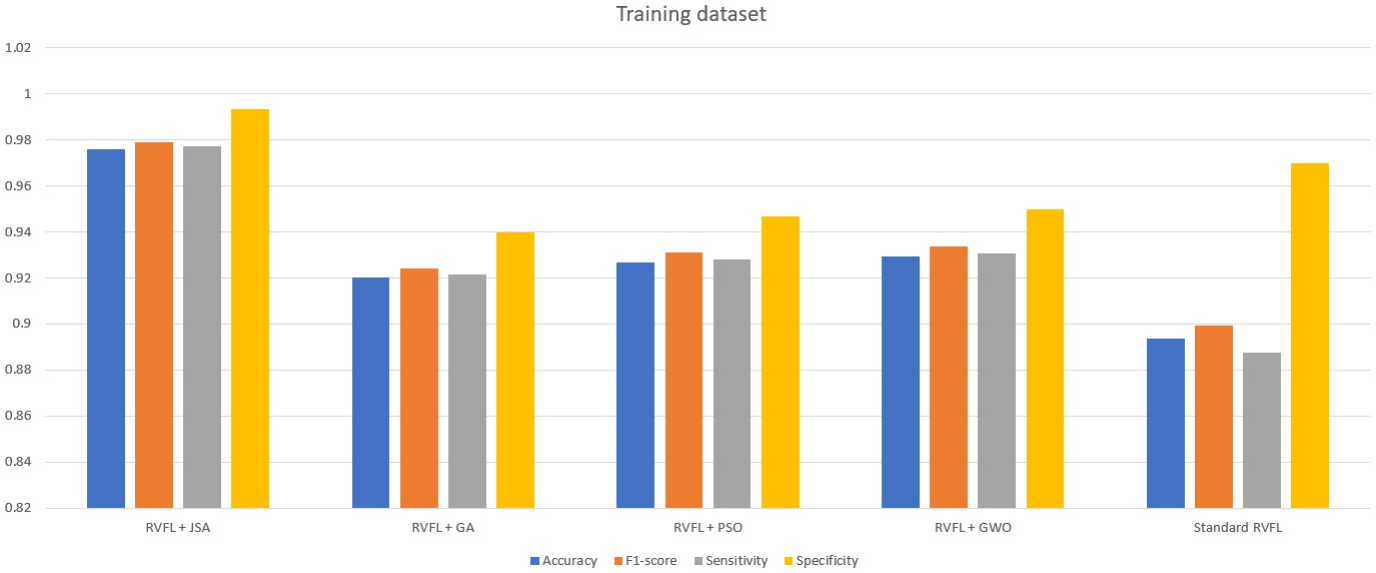
Accuracy, macro F1-score, sensitivity, and specificity for the training dataset.

**Fig 6:**
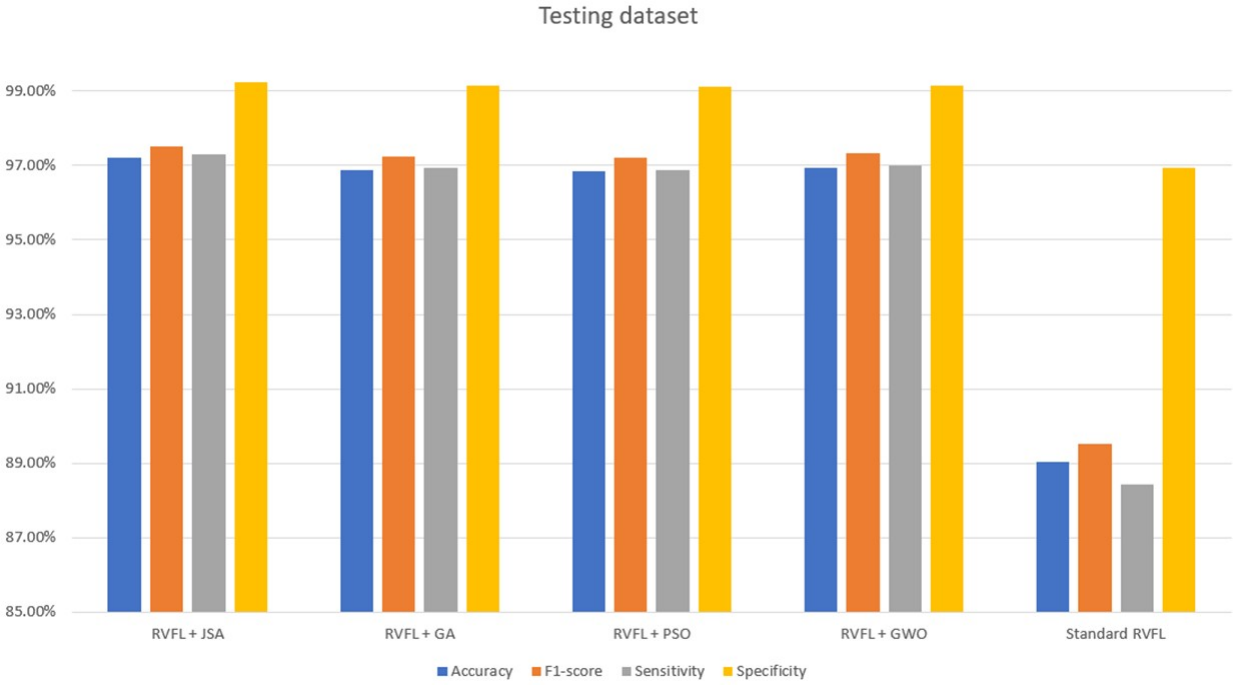
Accuracy, macro F1-score, sensitivity, and specificity for the training dataset.

**Fig 7:**
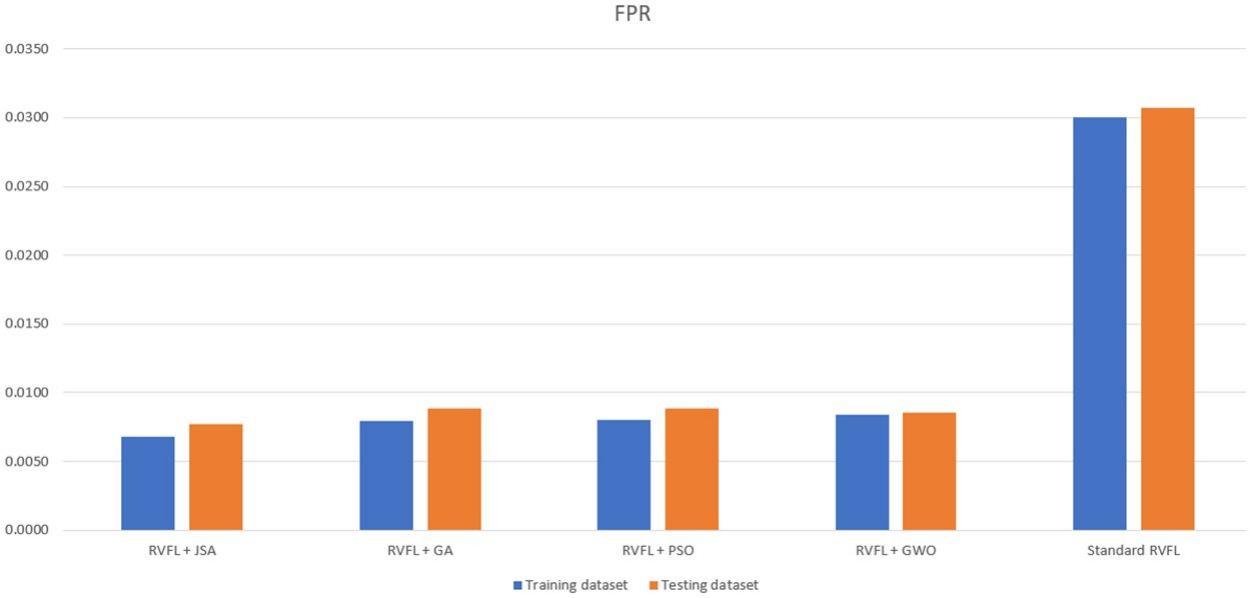
Comparison of false positive rates for the training and testing dataset.

**Fig 8:**
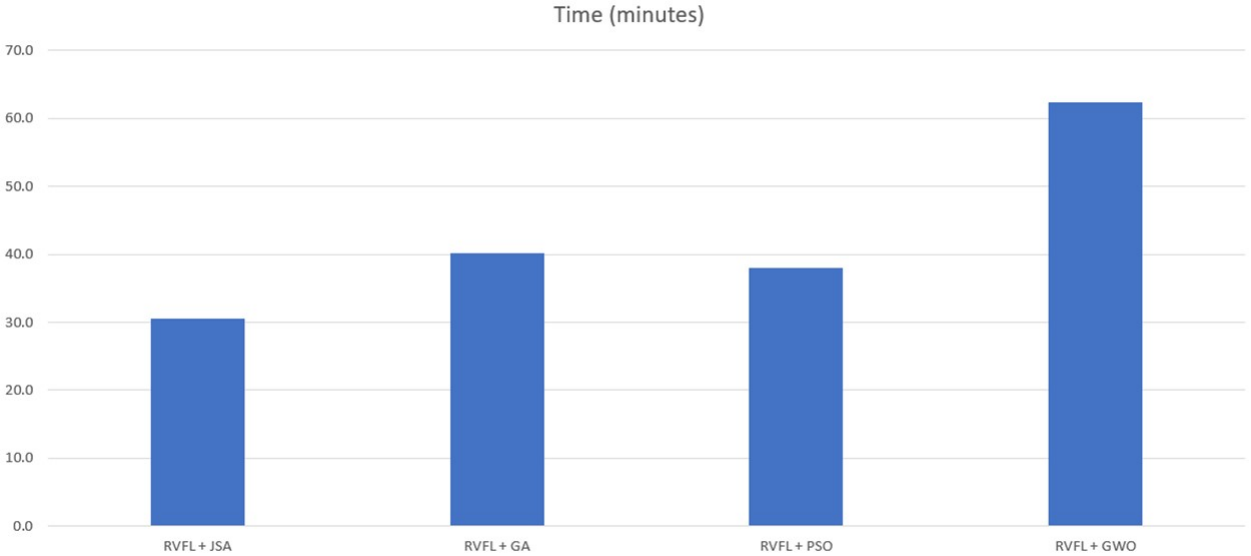
Time in minutes for each algorithm’s execution time.

As observed in Tables 6–11 and Figs 5–8, the RVFL+JS classification metrics (i.e., accuracy, F1-score, sensitivity, specificity and FPR) demonstrate that this method had the best performance results with the training and testing data, and required the minimal running time. This is a result of Jellyfish search algorithm’s design simplicity [31], showing that RVFL+JS is more effective than other metaheuristic algorithms.

### 5.6. Comparison of the same dataset with other works

The proposed model was compared with those of recent studies [24–26]. The previous experiments used the same dataset with the different models listed in Table 13.

**Table 13:** Accuracy comparison of other works to the proposed model.

Paper	Classifier	Best accuracy
Mahdavifar et al. (2020) [24]	Pseudo-Label Deep Neural Network	96.7%
AL-FAWA’REH et al. (2021) [25]	Random forest	96.4%
SAPUTRA et al. (2021) [26]	Convolutional Neural Networks	92.5%
Our approach	RVFL+JS	**98.41%**

**Fig 9:**
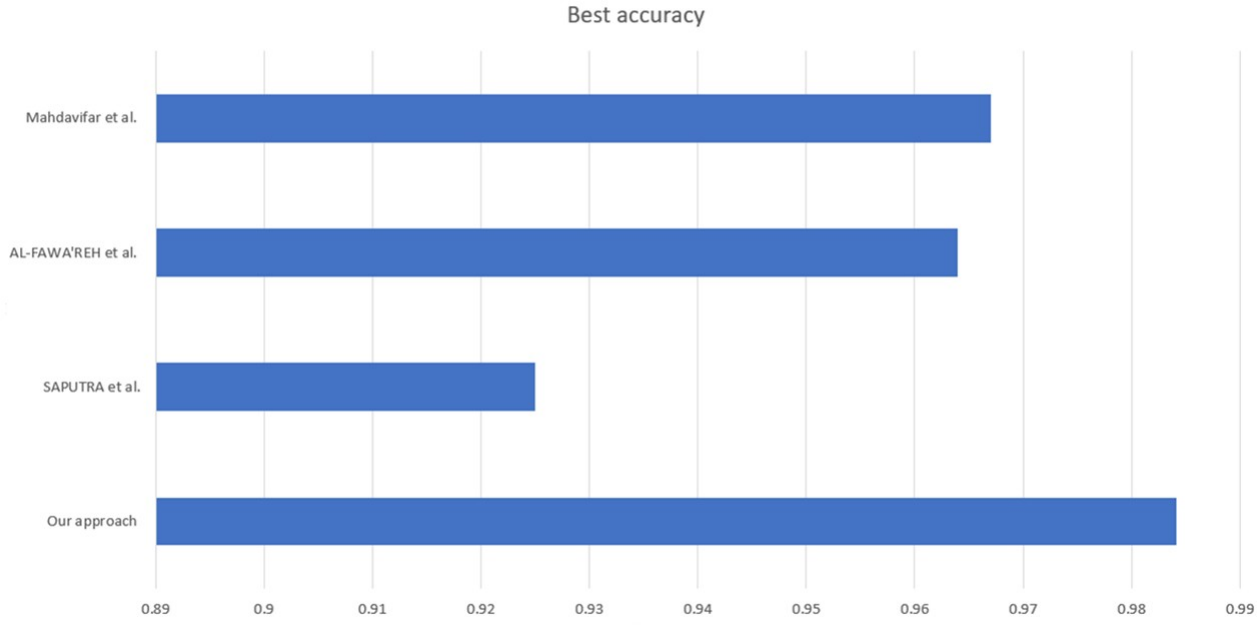
Accuracy comparison of other works to the proposed model.

The accuracy from [24] was 96.7%, from [25] was 96.4%, and from [26] was 95.0%. Fig 9 shows a comparison of the performance.

## 6. Conclusion and future work

Because of the rapid growth in the popularity of Android platform devices, hackers and attackers have a large playing field that is full of potential victims. Our work addresses this threat by examining the machine-learning platforms that are currently used to detect malware and other nefarious practices. We investigated Android application features and used the artificial JS optimizer to determine the effective features of malware detection.

Our work improves machine-learning efficiency, attaining a state-of-the-art malware detection accuracy of 98.41% while determining RVFL network hyperparameters that reduces runtime costs. In future research, the performance of our approach may be improved using additional machine-learning models. These can be compared with ready-to-use packages such as HyperOpt and Optuna frameworks to produce an automated tool for analyzing Android applications and generating a dynamically updated dataset. This will be beneficial to future researchers, as well as Android uses, as Android applications are published continuously and new security measures must be developed alongside them.

## Supporting information

S1 File (RAR)
